# First description of interdigital hyperplasia associated with contagious ovine digital dermatitis in two sheep

**DOI:** 10.3389/fvets.2022.1028880

**Published:** 2023-01-05

**Authors:** Maher Alsaaod, Robin Michael Schmid, Nathalie Zwahlen, Sara Soto, Nicole Wildi, Torsten Seuberlich, Adrian Steiner

**Affiliations:** ^1^Clinic for Ruminants, Department of Clinical Veterinary Science, Vetsuisse Faculty, University of Bern, Bern, Switzerland; ^2^Department of Infectious Diseases and Pathobiology, Vetsuisse Faculty, Institute of Animal Pathology, University of Bern, Bern, Switzerland; ^3^Division of Neurological Sciences, Vetsuisse Faculty Bern, University of Bern, Bern, Switzerland

**Keywords:** interdigital hyperplasia, sheep, contagious ovine digital dermatitis (CODD), *Treponema* spp., lameness

## Abstract

Interdigital hyperplasia (IH) is a fold of fibrous tissue protruding into the interdigital space that rarely occurs in sheep. Interdigital hyperplasia secondary infected with bovine digital dermatitis (BDD) treponemes has been reported in cattle in the course of the increasing spread of classical BDD lesions. In this report, we describe proliferative/ulcerative interdigital lesions associated with contagious ovine digital dermatitis (CODD) treponemes and clinically scored as (IH+CODD), occurring in both hind limbs of a ram and the left hindlimb of a ewe. Both cases exhibited epidermal hyperplasia, parakeratosis and focal-extensive areas of epidermal necrosis with numerous infiltrating neutrophils. *Treponema* PCR and fluorescence *in situ* hybridization (FISH) were positive for *Treponema* phylotype 1 (PT1). In addition, *Dichelobacter (D.) nodosus* and *Porphyromonas (P.) levii* were detected in the biopsy by PCR. In three slaughter sheep, without claw lesions, which were kept together with both affected sheep, *Treponema* spp. were detected neither with PCR nor FISH; the PCRs for *D. nodosus* and *P. levii* were also negative. Complete clinical healing occurred in the ewe within 6 weeks after three local applications of a chlortetracycline spray in 2 weeks intervals. This report is the first description of IH+CODD in sheep as demonstrated by a combination of histopathological and molecular analyses.

## Introduction

Interdigital hyperplasia (IH; interdigital fibroma; corn in cattle) is the outgrowth of skin folds from the junction of skin and horn in one or both claws and formation of hyperplastic interdigital skin of variable size located within the interdigital cleft (1–3). IH may have a heritable disposition in sheep and cattle, and predisposing factors in cattle may include stretching of the insertions of the distal interdigital ligaments, abnormally shaped hooves, animal overweight, bovine footrot, or slippery flooring (1, 4–6). IH is most commonly diagnosed in adult cattle and occasionally occurs in sheep and goats (2, 7). The degree of lameness depends on the size of the lesion and the presence of infection of the digital tissue. Ovine interdigital dermatitis (OID, foot scald) and bovine digital dermatitis (BDD) have been reported to occur concurrently with IH in sheep (3) and cattle (6, 8), respectively. OID is caused by *Fusobacterium necrophorum* (*F. necrophorum*) and often develops at both sides of the IH, causing further pain and lameness (3).

In cattle, several studies addressed the association between BDD and IH (9, 10). Since the wide spread of classical BDD lesions in the cattle population, superinfections of IH lesions with BDD-associated *Treponema* spp. (IH+BDD) have increased (11). *Treponema* spp., involved in BDD, are mainly of *Treponema medium, Treponema phagedenis* and *Treponema pedis* phylogroups (12, 13). These three phylogroups are also the major aetiological agents of contagious ovine digital dermatitis (CODD) (14–16). Other lameness-associated bacteria have also been isolated from CODD lesions; in particular, *Dichelobacter nodosus (D. nodosus)* and *F. necrophorum* (14, 17). *D. nodosus* is the primary etiological agent of ovine footrot and considered as a risk factor for CODD (14, 18).

This is the first report of IH+CODD in sheep, and it aimed at describing the clinical, histopathological and molecular findings in two clinical cases of IH+CODD.

## Case presentation

In January and February 2022, a ram (Texel, 5 years and 9 months) and a ewe (crossbreed, 6 years and 10 months) were both clinically inspected in the course of clinical footrot control. Both sheep were fixed in a claw chute and all limbs scored for footrot according to the Swiss Health Service for Small Ruminants adapted from Egerton and Roberts (19) using a scale from 0 (clinically healthy) to 5 (complete loss of the horn capsule). All limbs of the ram were scored as 2 (extensive interdigital dermatitis with involvement of the axial horn), except for the left forelimb that was scored as 3 (severe interdigital dermatitis and under-running of the horn of the heel and sole). Both hind limbs of the ewe were scored as 3. In addition, ulcerative lesions in the dorso-axial coronary band area of both claws of the left forelimb of the ewe were noticed during the examination and scored as CODD grade 1 according to Angell et al. (20). Lameness was scored according to Angell et al. (21), where 0 = sound and 3 = severely lame.

**Figure 1:**
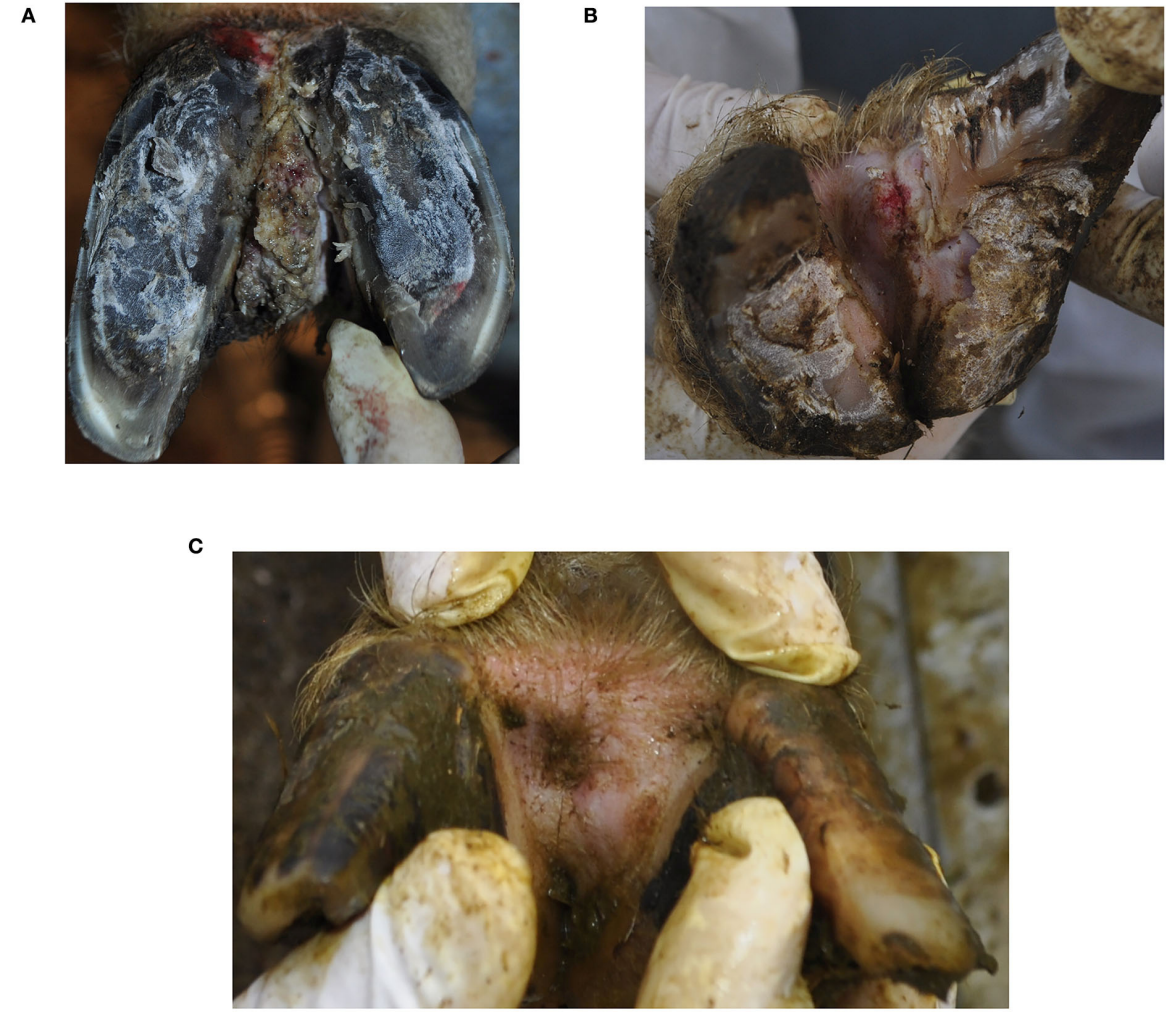
Clinical appearance of interdigital hyperplasia associated with contagious ovine digital dermatitis treponemes in a ram **(A)**, ewe **(B)** and after the clinical healing in the ewe **(C)**. The ewe **(B)** additionally showed signs of footrot (score 3).

Both hind limbs of the ram and the left hindlimb of the ewe additionally showed IH with proliferative and ulcerative lesions and were diagnosed clinically according to Fiedler et al. (6), Alsaaod et al. (8) and the International Committee for Animal Recording ICAR in cattle as IH+BDD (Figures 1A, B).

After disinfection of the skin with active chlorine (AquaJet^®^ Anolyte, Reichenburg SZ, Switzerland), an interdigital locoregional anesthesia at the level of the distal phalanx 1 was administered with 2% Lidocaine (Streuli Tiergesundheit AG, Uznach, Switzerland). After 10 min, one biopsy from the center of the lesion was collected using a sterile biopsy punch (4 mm in diameter with a maximum depth of 7 mm), and transferred directly to a sterile petri dish.

After biopsy sampling, chlortetracycline (Cyclospray, Dr. E. Graeub AG, Bern, Switzerland) was topically applied to the lesions without bandaging. The owner decided to slaughter the ram, and the ewe was further treated three times at intervals of 2 weeks with regular checks of the affected regions over a period of 2 months. The clinical healing of the CODD lesion occurred within 2 weeks and of IH+CODD within 6 weeks (Figure 1C).

Three slaughter sheep, which were kept together with both affected sheep but did not show any clinical signs of CODD or IH, served as negative controls. Two post-mortem biopsies were taken from each control sheep - one from the coronet and one from the skin of the interdigital cleft - and also transferred directly to a sterile petri dish. The control samples were pooled at animal level for PCR screening and separately evaluated by histopathology considering the most severe histopathological lesions.

Regular clinical inspections of the feet of both sheep were conducted within the framework of an ongoing footrot control program (animal experiment license no. SZ-34020).

## Histopathological and molecular biological analyses

Each individual biopsy was longitudinally sectioned. One half of each biopsy was used for PCR screening and the second half was fixed in 10% neutral buffered formalin for 24 h. These samples were then embedded in paraffin, cut into 4-μm sections and mounted on glass slides. The tissue sections were stained with hematoxylin and eosin (H&E) as well as silver stain (Warthin-Starry) for a better identification of spirochetes and were microscopically examined.

DNA was extracted from biopsy aliquots using a commercial DNeasy Blood and Tissue kit (Qiagen, Hilden, Germany) according to the manufactures instructions. PCR compatible quality of DNA isolates was confirmed by standard β-actin PCR as previously described (22). Subsequently, DNA extracts were screened for the presence of treponemal DNA using total-treponema PCR (TT-PCR) and nested specific PCR assays for *T. medium, T. phagedenis*, and *T. pedis*, and performed according to Moe et al. (23) and Evans et al. (24), respectively. All PCR tests were performed with the GoTaq^®^ Green Master Mix (Promega, Switzerland). All PCR products were separated by 1.5% agarose gel electrophoresis, with exception *P. levii* PCR with 2%.

Amplicon aliquots with positive TT-PCR and nested PCR (*T. phagedenis*) results were then gel-purified using NucleoSpin Gel and PCR Clean-up Kit (Marcherey-Nagel) according to the manufacturer's instructions and subjected to Sanger sequencing (Eurofins Genomics) using the same primer pairs as for the PCR reaction. The alignment of forward and reverse sequences and the identification of similar strains were performed using the NCBI BLAST webserver (https://blast.ncbi.nlm.nih.gov/Blast.cgi). To find the most similar strain, only the part of the sequence, matching in the alignment were used.

PCR for *D. nodosus* und *F. necrophorum* were performed according to Sullivan et al. (17). Primers for *P. levii* PCR were designed with Primer Blast (NCBI). The sequence of the forward primer (F-Primer 677) was 5′-AAGGCAGCTTACAAAAGTGTA-3′ and of the reverse primer (R-Primer 812) was 5′-TTTCGCTTGAGAGCATACAT-3′. The *P. levii* PCR parameters were as follows: 5 min at 95°C, 35 cycles (1 min, 95°C; 1 min, 54°C; 2 min, 72°C) and 5 min at 72°C. All primers are targeting 16S rRNA gene, with exception of hose for *F. necrophorum*, which leukotoxin (*lktA*).

Positive PCR results of *D. nodosus* were further analyzed by quantitative PCR (qPCR) according to Stauble et al. (25) with a cycle threshold (Ct) value of < 40 rated as positive. This qPCR distinguished between the protease genes *aprV2* and *aprB2*, thereby allowing the direct detection and differentiation of virulent and benign strains of *D. nodosus*, respectively.

For fluorescent *in situ* hybridization (FISH), serial 4-μm sections were prepared and hybridized as previously described by Rasmussen et al. (26). The oligonucleotide probes included probes specific for the genus *Treponema* and *Treponema* phylotype 1 (PT1). Both probes were 5'-labeled with the isothiocyanate derivative Cy3 (Eurofins Genomics, Ebersberg, Germany). The hybridization signal was scored from 0 to 3 according to Klitgaard et al. (27): 0 = no hybridization, 1 = sparse hybridization, 2 = moderate hybridization, and 3 = strong hybridization.

## Results

Clinical examination revealed a large and bilateral IH with chronic proliferative lesions of the tissue covering the whole IH in the ram, while a small IH with an acute ulcerative lesion on the top of it was present in the ewe (Figures 1A, B). Both sheep showed uneven steps and were scored as mildly lame (score =1), as affected limbs were not clearly identifiable at locomotion (score = 1).

**Figure 2:**
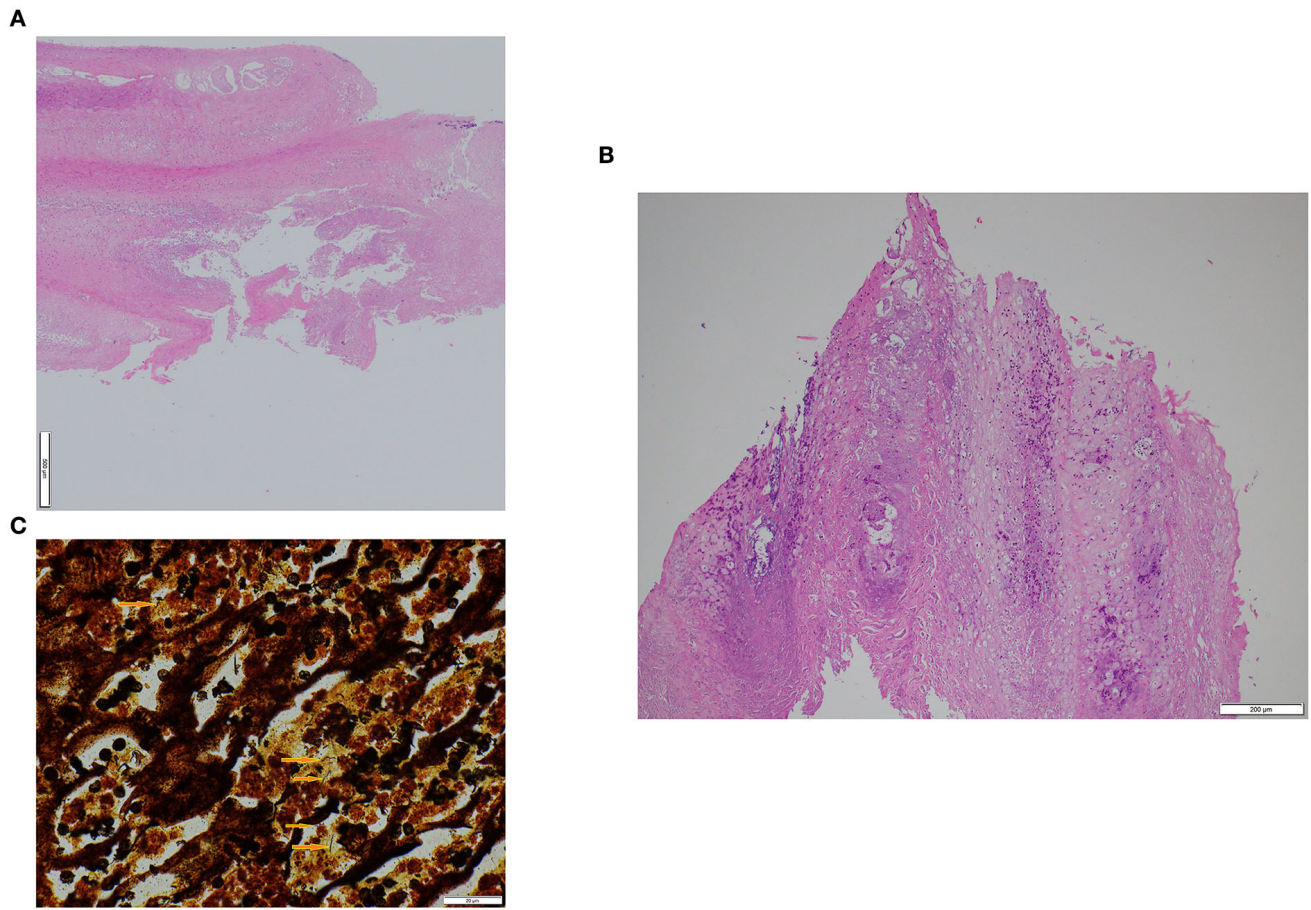
Histological appearance of interdigital hyperplasia associated with contagious ovine digital dermatitis treponemes in a ram **(A)** and a ewe **(B)**. Warthin-Starry staining showing helicoidal-shaped bacteria (arrows) compatible with spirochetes in the ram **(C)**. Magnification (100 ×).

The histopathological findings were similar in the ewe and ram samples (Figures 2A, B). The epidermis was markedly hyperplastic and partly hyperkeratotic (mainly parakeratosis) with focal-extensive areas of epidermal necrosis with presence of numerous neutrophils. In the nearby epidermis, part of the keratinocytes displayed prominent cellular degeneration. Bacterial colonies of variable shape were present on the sample's surface. Rather numerous lymphocytes and plasma cells as well as some neutrophils and rarely eosinophils infiltrated the dermis. In both cases, helicoidal-shaped bacteria compatible with spirochetes were observed in the necrotic areas on the epidermal surface and between the keratinocytes, made visible with the Warthin-Starry-stain (Figure 2C). In the samples of the control animals, only mild to moderate epidermal hyperplasia and mild lymphoplasmacytic dermal inflammation were observed.

The DNA samples of the two biopsies originating from the ram and the ewe tested positive by the TT-PCR and the nested PCR for *T. phagedenis* (*T. medium* and *T. pedis* were not detectable). The obtained nucleotide sequences of the two PCR products of each biopsy originating from the ram and ewe, respectively, showed an identity of 100 and 95.29% to *Treponema* PT1 (Accession number AM942445.1) for the TT-PCR product and 95.92 and 97.27% to *Treponema* PT1 (Accession number AM942445.1) for the nested PCR product.

In addition to *Treponema* PT1, *D. nodosus* and *P. levii* were detected in both biopsies; while the PCR for *F. nechrophorum* remained negative. The subsequent qPCR for *D. nodosus* was positive for the virulent strain of *D. nodosus* (Ct values = 24.5 and 24 for two biopsies originated from the ram and ewe, respectively).

The samples of the control biopsies of the three clinically healthy sheep were negative for *Treponema* spp. (TT-PCR and nested-PCR), *D. nodosus, F. necrophorum*, and *P. levii*.

**Figure 3:**
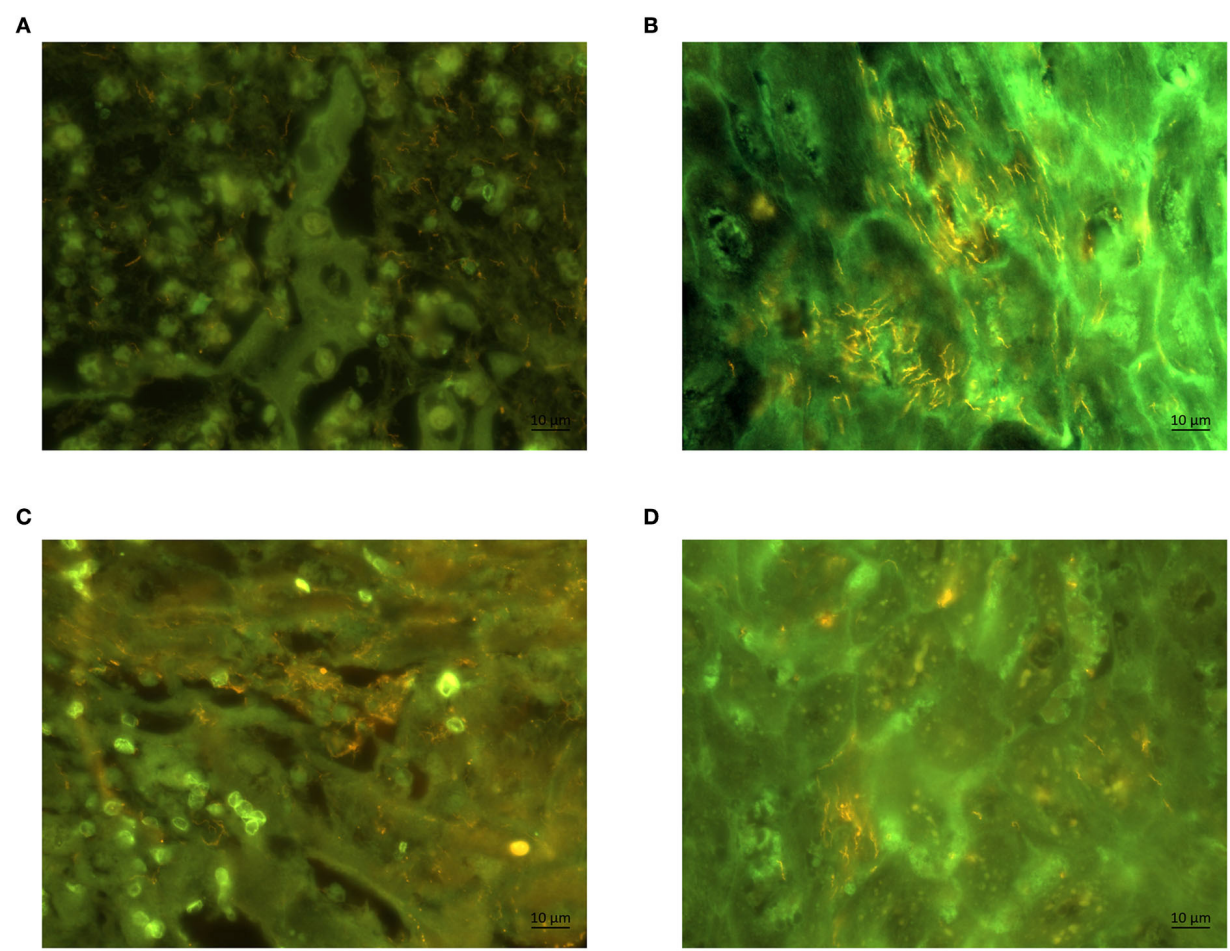
Fluorescent *in situ* hybridization of biopsies of a ram **(A)** and a ewe **(B)** with probes for genus *Treponema* (Cy3 labeled). Section of the same biopsy hybridized with (Cy3 labeled) species specific oligonucleotide probe for *Treponema* phylotype PT1 in a ram **(C)** and ewe **(D)**. The *Treponema* organisms appear yellow; magnification (60 ×).

By FISH, the general oligonucleotide probe for *Treponema* spp. revealed a severe, extensive treponemal epidermal infiltration (score 3) in both biopsies of affected ram and ewe (Figures 3A, B). Additional FISH analysis with the *Treponema* PT1 specific oligonucleotide probe revealed a strong hybridization (score 3) (Figures 3C, D), again in both biopsies of affected ram and ewe. All biopsies originating from three control sheep were tested negative for *Treponem*a spp by FISH.

## Discussion

According to the best of the authors ' knowledge, this is the first report describing IH associated with CODD in sheep. *Treponema* spp. were found in the proliferative/ulcerative lesions of the hyperplastic interdigital tissue samples, and this was confirmed by histopathological and molecular analyses. In contrast, neither PCR nor FISH allowed to detect *Treponema* spp. in the control animals.

OID (foot scald) is caused by *F. necrophorum* (3) and its absence in both cases excluded the etiology of OID in our report. Both sheep were affected with ovine footrot caused by *D. nodosus*. Footrot is considered as a risk factor for CODD (14, 28). Several studies have investigated the association of *Treponema* spp. with CODD lesions (14–16) and hypothesized that CODD was derived from BDD lesions and may have crossed species barrier, primarily by co-grazing with cattle (29). CODD is a progressive infectious foot disease, which presents as erosion/ulceration at the coronary band and terminates in avulsion of the entire hoof capsule (20). Infection and transmission routes of *Treponema* spp. in both described cases are not known, as co-grazing was not practiced in this farm. The introduction of CODD treponemes by purchase of carriers is suspected.

Parakeratotic hyperkeratosis and spirochetal colonization in the necrotic areas on the epidermal surface and between the keratinocytes were the most prominent histopathological features in both cases. These findings are consistent with the typical histological features of BDD (30, 31).

The nested PCR for *Treponema* species according to Evans et al. (24) was positive for *T. phagedenis* with bands of the respective size (400 bp) and negative for *T. medium* and *T. pedis*. Sequence analysis of both PCR products (*T. phagedenis*) from two biopsies showed that these were not species-specific. Indeed, they matched sequences derived from the yet uncultured *Treponema* PT1. This indicates, that *Treponema* species other than *T. phagedenis* are present in both biopsy cases of IH+CODD and the applied nested PCR is not specific enough for *Treponema* spp. in sheep. Similar observations were found by sequencing *Treponema* spp. associated DD lesions, positive for *T. phagedenis* of captive European bison (32). The 16S rRNA gene used as target is highly conserved and present in all bacteria. This might explain this phenomenon.

*Treponema* PT1 is the most prevalent *Treponema* spp. in BDD lesions (95%), invading deep into the stratum spinosum (27). As the control biopsy samples tested negative for the known foot-infection associated bacteria, it is suggested that *Treponema* spp. are involved as primary bacterial pathogens in the described IH+CODD lesions. *P. levii* is a well-known opportunistic pathogen and has been already isolated from ID and BDD lesions in cattle (33, 34). In a previous study, the role of *P. levii* in influencing the overall metabolic processes of the BDD microbiota was demonstrated (35).

IH is characterized by a fold of fibrous tissue protruding into the interdigital space. It represents an uncommon foot disease in sheep. Just under 1% of the Swedish slaughter lambs showed IH in the footrot prevalence study by Konig et al. (7). IH may be hereditary; particularly affected rams should not be bred (3). In our report, only the ewe with the less severe lesions was treated with topical administration of chlortetracycline. Treatment with chlortetracycline revealed effective to treat CODD (36). Large lesions of IH causing chronic lameness can be surgically removed under local anesthesia (37) or may be treated with salicylic acid as successfully performed in cattle (8).

## Conclusion

This report is the first description of IH+CODD lesions in sheep characterized by the presence of *Treponema* spp. in proliferative and ulcerative tissue covering IH lesions. It was confirmed by clinical, histopathological and by molecular biological analyses. *Treponema* (PT1), *D. nodosus* and *P. levii* and typical histological changes including parakeratotic hyperkeratosis and spirochetal colonization were identified. IH+CODD should be considered for the differential diagnosis of interdigital lesions in sheep.

## Data availability statement

The original contributions presented in the study are included in the article/supplementary material, further inquiries can be directed to the corresponding author.

## Ethics statement

The animal study was reviewed and approved by animal experiment license no. SZ-34020. Written informed consent was obtained from the owners for the participation of their animals in this study.

## Author contributions

MA was responsible for data collection, molecular analyses, and writing the first draft of the manuscript. RS and NZ supported the data collection. SS performed the histological analyses. NW and TS supported the data analyses. AS edited the manuscript and supervised the study. All authors contributed to the manuscript and approved the final version.
